# Computational Modeling Approach to Profile Hemodynamical Behavior in a Healthy Aorta

**DOI:** 10.3390/bioengineering11090914

**Published:** 2024-09-12

**Authors:** Ahmed M. Al-Jumaily, Mohammad Al-Rawi, Djelloul Belkacemi, Radu Andy Sascău, Cristian Stătescu, Florin-Emilian Țurcanu, Larisa Anghel

**Affiliations:** 1 Institute of Biomedical Technologies, Auckland University of Technology, Auckland 1010, New Zealand; 2 Center for Engineering and Industrial Design, Waikato Institute of Technology, Hamilton 3240, New Zealand; mohammad.al-rawi@wintec.ac.nz; 3 Faculty of Engineering, Chemical and Materials Engineering, The University of Auckland, Auckland 1010, New Zealand; 4 Unité de Développement des Equipements Solaires, UDES, Centre de Développement des Energies Renouvelables, CDER, Tipaza 42004, Algeria; belkacemidjelloul@gmail.com; 5 Internal Medicine Department, Grigore T. Popa University of Medicine and Pharmacy, 700503 Iași, Romania; radu.sascau@umfiasi.ro (R.A.S.); cristian.statescu@umfiasi.ro (C.S.); larisa.anghel@umfiasi.ro (L.A.); 6 Cardiology Department, Cardiovascular Diseases Institute, Prof. Dr. George I.M. Georgescu, 700503 Iași, Romania; 7 Building Services Department, Faculty of Civil Engineering and Building Services, Gheorghe Asachi Technical University, 700050 Iaşi, Romania; florin-emilian.turcanu@academic.tuiasi.ro

**Keywords:** tetrahedral mesh, polyhedral mesh, healthy aorta, computational fluid dynamics, endothelial cell activation potential

## Abstract

Cardiovascular diseases (CVD) remain the leading cause of mortality among older adults. Early detection is critical as the prognosis for advanced-stage CVD is often poor. Consequently, non-invasive diagnostic tools that can assess hemodynamic function, particularly of the aorta, are essential. Computational fluid dynamics (CFD) has emerged as a promising method for simulating cardiovascular dynamics efficiently and cost-effectively, using increasingly accessible computational resources. This study developed a CFD model to assess the aorta geometry using tetrahedral and polyhedral meshes. A healthy aorta was modeled with mesh sizes ranging from 0.2 to 1 mm. Key hemodynamic parameters, including blood pressure waveform, pressure difference, wall shear stress (WSS), and associated wall parameters like relative residence time (RRT), oscillatory shear index (OSI), and endothelial cell activation potential (ECAP) were evaluated. The performance of the CFD simulations, focusing on accuracy and processing time, was assessed to determine clinical viability. The CFD model demonstrated clinically acceptable results, achieving over 95% accuracy while reducing simulation time by up to 54%. The entire simulation process, from image construction to the post-processing of results, was completed in under 120 min. Both mesh types (tetrahedral and polyhedral) provided reliable outputs for hemodynamic analysis. This study provides a novel demonstration of the impact of mesh type in obtaining accurate hemodynamic data, quickly and efficiently, using CFD simulations for non-invasive aortic assessments. The method is particularly beneficial for routine check-ups, offering improved diagnostics for populations with limited healthcare access or higher cardiovascular disease risk.

## 1. Introduction

Early diagnosis of cardiovascular diseases (CVD) is a key area of research across medical, engineering, and biological sciences, driven by the need to mitigate the high mortality associated with late-stage disease [1]. Advanced computational techniques are increasingly utilized to enhance the accuracy of disease modeling based on patient-specific variables. For instance, two-dimensional (2D) clinical imaging modalities, such as magnetic resonance imaging (MRI) and computed tomography (CT) scans, can be transformed into detailed three-dimensional (3D) models. These 3D models allow for intensive computational analysis of hemodynamic parameters, including arterial wall mechanics and blood flow characteristics, providing critical insights into the pathophysiology of CVD at an individual level [2]. The hemodynamic behavior of the aorta plays a crucial role in cardiovascular health, serving as the main conduit for blood flow from the heart to the systemic circulation. Assessing the hemodynamic parameters non-invasively has become a critical aspect of early diagnosis and management of cardiovascular diseases [3]. Advanced imaging techniques, such as 4D-flow MRI, combined with computational fluid dynamics (CFD), enable precise evaluation of these parameters, offering valuable insights into the cardiovascular health of patients. By comparing patient-specific data to normative values derived from healthy aortic behavior, clinicians can identify early signs of disease, allowing for timely intervention and potentially improving patient outcomes [2,3].

The importance of early detection is underscored by the severe consequences of advanced CVD, which often leads to irreversible damage to the arteries and heart [2,3,4,5]. CVD remains the leading cause of death in developed nations. In the United States, for example, a person dies from CVD every 33 s, contributing to approximately 697,000 deaths annually, which accounts for 1 in every 5 deaths [1]. Similarly, in Europe, CVD is responsible for over 3.9 million deaths each year, representing 45% of all deaths in the region, with coronary heart disease and stroke being the most prevalent causes [4,5].

In Australia, over 500,000 individuals are currently living with heart disease, with approximately 57,000 heart attacks occurring each year. New Zealand reports that CVD causes one death every 90 min, highlighting the pervasive nature of the disease [6,7,8,9,10,11]. These statistics underscore the urgent need for improved diagnostic strategies, particularly non-invasive methods that can detect CVD in its earliest stages, thereby enabling timely intervention and reducing the overall disease burden.

Research efforts continue to focus on refining these diagnostic tools, with significant advancements being made in understanding the progression of CVD. Computational fluid dynamics (CFD) has emerged as a powerful tool in simulating blood flow and pressure within arteries, contributing to the identification of critical regions prone to atherosclerosis and other cardiovascular conditions [2,12]. These innovations hold promise for more personalized and effective management of CVD, potentially transforming the approach to cardiovascular care in the coming years.

CFD analysis has been used for the past two decades to simulate CVD progression under a variety of initial conditions, with ongoing improvements to the models as the software and processor speeds increase in sophistication. CFD assesses the influence of a variety of arterial characteristics, such as wall stiffness and lumen diameter, inter alia, on the progression of different forms of CVD, including aneurysm and arterial stenosis [12,13,14,15,16]. 

CFD analysis takes precise clinical data from 2D images that relate to specific cases and other measures, such as blood pressure and velocity waveforms, to validate the arterial rheological characteristics during the disease development [2]. Employing contrast-enhanced computed tomography (CTA) scan images, 2D segments are generated, which then develop the 3D geometry that can be analyzed using fluid dynamics mathematical models [17]. The main equations used in research on arterial CFD modeling for blood flow in arteries are the continuity equation and the momentum equation governing the arterial wall structure [18,19,20,21,22]. The fluid dynamics equations require input and output data (boundary conditions); consequently, phase-contrast Cardiovascular Magnetic Resonance Imaging (CMRI) provides these boundary conditions, with the magnitude of the velocity at the peak systole used to solve the computational domain [23] as a steady-state case. 

**Figure 1:**
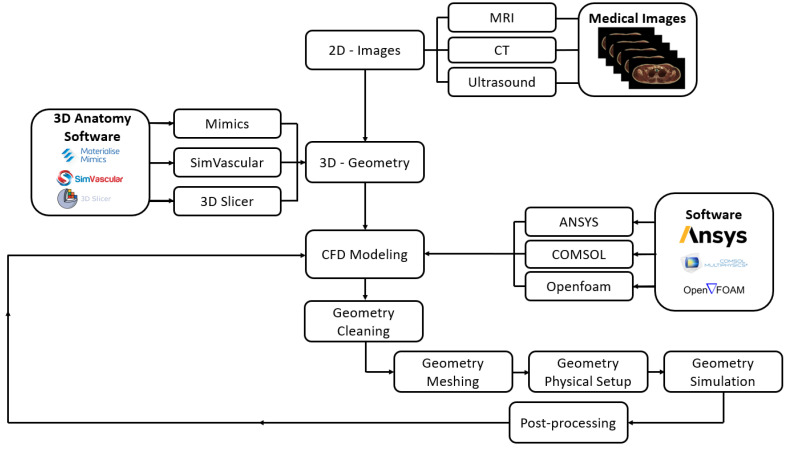
The current processes used for CFD simulation.

The processes used for CFD modeling are outlined in the flow chart shown in Figure 1. One of the key processes required to achieve robust CFD results is the mesh quality, and all of the computational analysis literature must provide evidence to validate their results based on the mesh element size used. In general, the finer the mesh, the better the quality of the results; however, a very fine mesh generates a high number of elements, which lengthens the simulation time, requiring advanced software packages and high-performance computers to run the simulations. 

The aorta geometry is constructed with a Non-Uniform Rational B-Splines (NURBS), 3D organic free-form solid from the 2D lines, circles, arcs, and curves generated by the software package as a Standard Triangle Language (STL) file [23,24]. In many cases, along with the 2D images, useful clinical boundary conditions are provided by the equipment, such as blood flow and pressure waveforms, which are required for the CFD modeling setup [2].

Finding a suitable mesh is a crucial process in obtaining accurate CFD models of hemodynamic behavior. The aortic geometry can be meshed using different methods and element sizes across several different CFD software packages. Simão et al. [24] used CFD COMSOL-Multiphysics to simulate the aorta to evaluate the wall shear stress (WSS) and vortices over time using a tetrahedral mesh for three different patients. Their study reached convergence with the following mesh elements: 225,056 for a healthy aorta, and 242,859 for an unhealthy aorta, with, respectively, 0.1–0.55, 0.1–0.35, and 0.1–0.6 m/s velocity ranges and 2–14 (0.015–0.105 mmHg), 1–7 Pa (0.075–0.052 mmHg), 0–14 Pa (0–0.105 mmHg) WSS ranges for the aortic geometries. Petuchova et al. [25] studied two aortic geometries using SimVascular software, obtaining a WSS range between 0.3 and 0.6 Pa (0.0022–0.0045 mmHg). Their mesh was performed using the TetGen meshing method, which reached 67,571 elements. However, the authors noted that the software used had limitations, identifying some distortion in hemodynamic parameters such as the oscillatory shear index (OSI), which is used to identify the mechanical factors of stress fluctuations resulting from the blood flow per cardiac cycle.

The WSS is a pivotal hemodynamic parameter in the study of aortic pathophysiology as it directly influences the health and function of the aortic endothelium. In the aorta, WSS plays a crucial role in the initiation and progression of various cardiovascular diseases, including aortic aneurysms, dissections, and atherosclerosis [2,26].

The aorta, being the largest artery in the body, is subjected to high and varying flow velocities, especially during systole. The distribution of WSS within the aorta is non-uniform, with elevated levels typically observed in regions of curvature, such as the aortic arch and at bifurcations, where complex flow patterns and secondary flows are common. These regions of high WSS are often associated with endothelial stress, which can lead to cellular damage and contribute to the development of aortic pathologies. Low WSS, on the other hand, is often found in areas of flow recirculation or stagnation, such as the inner curvature of the aortic arch or downstream of stenotic lesions. These areas are particularly prone to atherosclerotic plaque formation, as low WSS is associated with an inflammatory endothelial phenotype, increased permeability to lipids, and a pro-atherogenic environment [27].

Accurately modeling WSS in the aorta is therefore critical for understanding the underlying mechanisms of aortic diseases and for predicting sites at risk of pathological changes. CFD models, which simulate blood flow and its interaction with the aortic wall, are invaluable tools in this regard [28].

Given the clinical importance of WSS in predicting aortic diseases, such as the formation or rupture of aneurysms and the progression of aortic atherosclerosis, the selection of an appropriate mesh and simulation approach is critical. Accurate WSS assessment can help identify individuals at high risk for these conditions, enabling early intervention and improving clinical outcomes [29]. Therefore, continued advancements in CFD modeling and mesh optimization are essential for enhancing our understanding and management of aortic diseases.

Osswald et al. [30] presented CFD results for aortic geometry using Star-CCM+ with mesh elements numbering from 210,000 to 420,000, treating the blood as having Newtonian properties and laminar flow. They found that there was high WSS located at the entry tear compared to the surrounding wall. Meng et al. [31] used ANSYS 2022R1-Fluent to study the aorta. Their mesh independence test aimed to find the correct element mesh size. They use a 0.15 mm Prism boundary layer with a 1.2 growth ratio at the boundary to calculate high blood velocities’ changes near the aortic wall. Their results showed high OSI and wall shear stress in an enlarged ascending thoracic aortic aneurysm. 

Qu et al. [32] discussed the importance of the mesh independence test for their computational modeling of the blood flow in the portal vein under different grafts to correlate between the pressure and ascites drainage. They found that, upon doubling the mesh element numbers to 440,000, the pressure value increases by 4% within the acceptable range of the pressure drop. Caballero and Laín [33] surveyed several mesh independence studies relating to the aorta. They identified the importance of mesh parameters for quantifying the difference associated with spatial discretization, highlighting how essential this step is to effective computational modeling. 

Numata et al. [14] used ANSYS-ICEM CFD to generate a tetrahedral mesh model containing more than two million elements, having three boundary layers near the artery wall to investigate the flow velocity and wall shear stress (WSS) in peak systole using ANSYS-Fluent. Their results, in a healthy aorta, show a turbulent flow in the aortic arch that generates high WSS contours at the bifurcation. Kumar et al. [34] presented three tetrahedral meshes for aortic geometry assessed using steady-state analysis to test the mesh sensitivity with respect to WSS values. Their results showed a difference of 30.45% between the coarse and fine mesh, highlighting the degree to which mesh choice can impact the results of the aorta model. Zhu et al. [21] used automatic meshing under ANSYS ICEM with a tetrahedral mesh, three boundary layers near the wall, and a total element count between one and two million. The results show the WSS contours reached 19.6 Pa (0.15 mmHg) at the bifurcation and around 10 Pa (0.08 mmHg) at the aortic arch. In our previous study [15], we used a tetrahedral mesh with an element number of close to five million to assess short and long branches using non-Newtonian power law for blood properties. Our results showed that the time-averaged wall shear stress (TAWSS) at the aortic arch was around 8 Pa (0.06 mmHg). Spiegel et al. [35] compared the tetrahedral and the polyhedral mesh types on the cerebral artery in the presence of an aneurysm. Their results showed that the polyhedral mesh achieved convergence and homogeneity faster than the tetrahedral mesh using ANSYS-Fluent with the assumption of Newtonian blood properties (density 1050 kg/m^3^ and viscosity 0.004 Pa.s). They noted that polyhedral meshes were preferable for cerebral aneurysms, having an uncertainty between 3 and 6% and a shorter computation time; however, the WSS results may be more precise with a tetrahedral mesh. 

The aim of this study is to find the model that obtains accurate results whilst reducing the simulation time by adjusting the mesh type (tetrahedral or polyhedral) and element size in such a way that does not impact the quality of the model’s representation of the hemodynamic properties of the aorta. The benefit of this study will be to provide proof of concept of a procedure to assist in the development of a non-invasive assessment of a patient’s cardiovascular health, able to be performed by a medical practitioner with non-specialized computer equipment during a normal check-up.

## 2. Materials and Methods

This study compares the tetrahedral and polyhedral mesh methods using the laminar flow regime, inputting the blood flow and pressure waveforms as boundary conditions and treating the blood as non-Newtonian, with a rigid artery wall and no-slip boundary layer.

### 2.1. Aortic Geometry and Boundary Conditions

**Figure 2:**
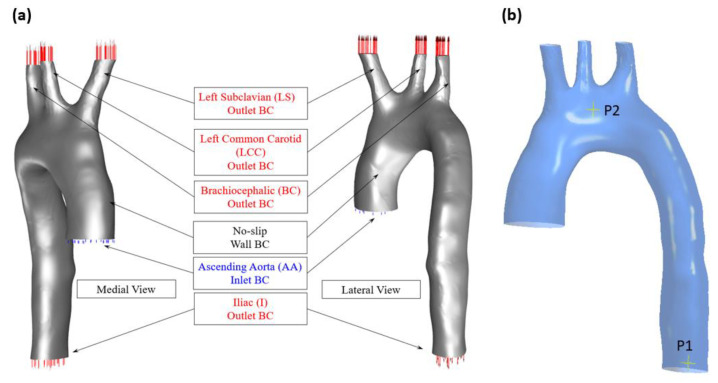
(**a**) The aortic geometry showing the lateral and medial views with the boundary conditions and the computational domain; (**b**) the location of the points considered for investigation (adapted from [2]).

To construct the aortic geometry, 2D MRI images were converted to a 3D STL file, which was then exported into ANSYS-Fluent. The STL geometry was modified using Design Modeler (DM) given its non-uniform shape, or NURBS geometry, which is illustrated in Figure 2. The aortic geometry consists of the following aortic branches: the left subclavian (LS), the left common carotid (LCC), and the brachiocephalic (BC). These bifurcations were treated as outlets with boundary conditions defined using relevant pressure waveforms, as provided in Table 1. Their known cross-sectional areas with equivalent diameters are also listed in Table 1, along with the boundary conditions for the ascending aorta and iliac artery. The boundary conditions and geometry used in this study were obtained from patient data in accordance with appropriate ethical protocols (approval NTX/09/11/109) provided by the Institute of Biomedical Technologies as published in our previous study [2].

### 2.2. CFD Setup

The governing equations—which are activated in ANSYS Fluent—are (1) the continuity equation, and (2) the momentum in terms of the Navier–Stokes equation. These solve the problem as pulsatile flow and pressure waveforms with non-Newtonian blood properties and are provided below.

(1) $$\frac{\partial \rho }{\partial t}+\nabla \cdot \left(\rho \overset{⃑}{v}\right)=0$$

(2) $$\rho (\frac{d\overset{⃑}{v}}{dt}+\overset{⃑}{v}\cdot \nabla \overset{⃑}{v})=-\nabla p+\mu (\dot{\gamma })\nabla^{2}\overset{⃑}{v}+\overset{⃑}{f}$$

where the blood density is assumed at 1060 kg/m^3^ (ρ), treated as incompressible pulsatile blood flow solved in the three-dimensional x, y, and z space; therefore, Equation (1) can be expressed as $\nabla \cdot \left(\overset{⃑}{v}\right)=0$. In this study, the pressure waveforms with respect to ∇p in mmHg are shown in Table 1, and the viscosity coefficient, μ, is a function of the shear rate as the blood becomes less viscous as the shear rate increases. These equations are solved with the assumption that the pulsatile flow is transitioning from laminar to turbulent flow and adheres closely to laminar flow using the Navier–Stokes equations, whilst also assuming the blood is non-Newtonian using the Carreau model [36]. This is set up within ANSYS Fluent to consider the blood’s shear-thinning behavior using Equation (3).

(3) $$\mu =\mu_{\infty }+\left(\mu_{o}-\mu_{\infty }\right){\left[1+{\left(\lambda \dot{\gamma }\right)}^{2}\right]}^{\frac{n-1}{2}}\;$$

where the coefficients $\mu_{\infty }$ and $\mu_{o}$ are the infinite and zero shear viscosities kg/(m/s) with the following values, 0.0035 and 0.056, respectively. These coefficients are asymptotic, which depend on the lowest shear rates, corresponding to the lowest pressure drop in the artery flow as well as higher shear rates [36]. The parameters λ=3.313 and n=0.568 are the time constant in (s) and the power-law index, respectively. The main objective of this equation is to determine the strain rate,$\;\dot{\gamma }$, in 1/s to determine the blood viscosity in kg/(m/s) [37]. The rationale for using a non-Newtonian model is to develop a model with a minimum time for the simulation to produce robust results for assessing the risk of cardiovascular diseases (CVDs) via the aortic geometry. This approach to blood properties aligns with prior research on early diagnosis of CVDs using computational methods, including identifying atherosclerosis at the descending aorta [2], aneurysms at the ascending aorta, aortic dissection [38], and abdominal aortic aneurysms [22]. It used the Carreau model to predict any abnormalities of the WSS contours. Therefore, this model is effective for considering the aortic blood flow circulation and reverse flow within the changes to the geometrical structure, such as the large degree of curvature at the aortic arch, bifurcation, and branching.

**Figure A1:**
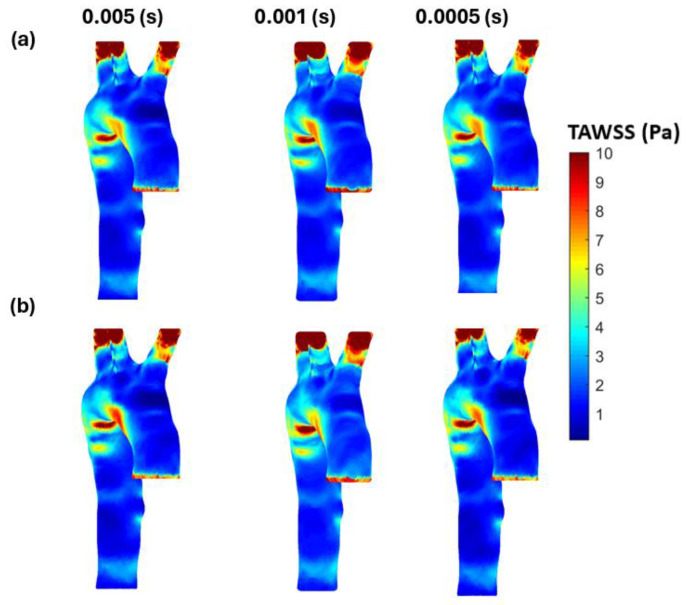
The TAWSS for the three-time steps (0.005, 0.001, and 0.0005 s) for 0.4 mm mesh element size: (**a**) polyhedral mesh; and (**b**) tetrahedral mesh.

**Figure A2:**
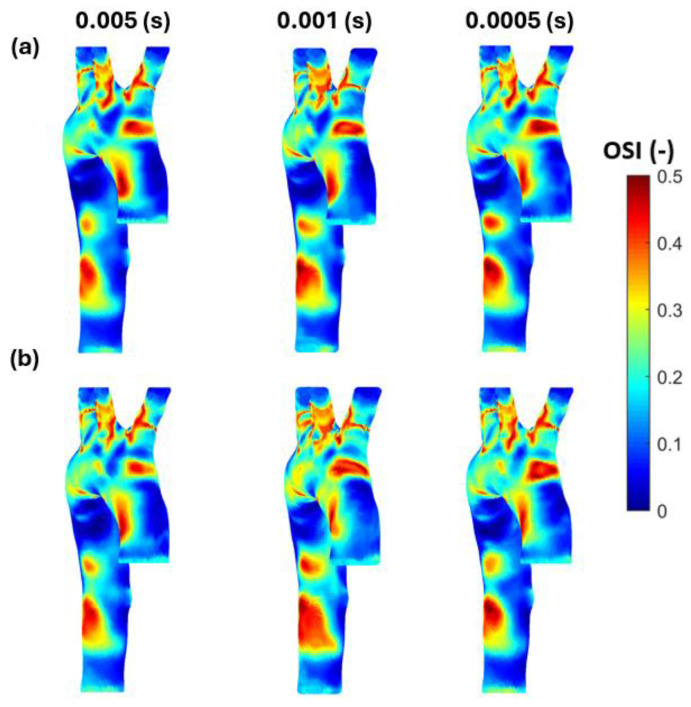
The OSI for the three-time steps (0.005, 0.001, and 0.0005 s) for 0.4 mm mesh element size: (**a**) polyhedral mesh; and (**b**) tetrahedral mesh.

**Figure A3:**
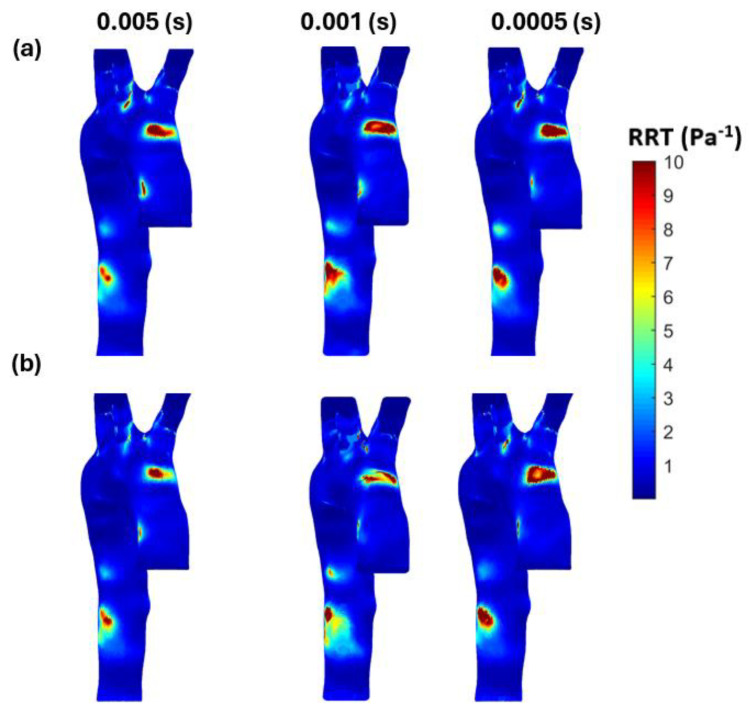
The RRT for the three-time steps (0.005, 0.001, and 0.0005 s) for 0.4 mm mesh element size: (**a**) polyhedral mesh; and (**b**) tetrahedral mesh.

The computational model was set using a second-order upwind scheme for spatial discretization and the SIMPLE algorithm (Semi-Implicit Method for Pressure-Linked Equations) for pressure-velocity coupling. Three cardiac cycles were simulated to minimize the influence of initial conditions, and the last cycle was considered in our results as the results become asymptotic after the second cycle. For the run calculation, we set the number of time steps at 3000 with a time step size of 0.001 s and a maximum number of iterations of 30. The selected time step size was compared against 0.005 and 0.0005 s to ensure that the results for the investigated parameters showed a negligible difference (see, respectively, Figure A1, Figure A2 and Figure A3 in Appendix A). The convergence criteria for the solutions were considered when the residuals for the continuity and the velocity equations were achieved at $1\times 10^{-5}$ [37,38]. These simulations used a stationary wall and no-slip shear condition. The processor used for this simulation is a readily available retail model consisting of an Intel^®^ Core™ i7-8750H CPU @ 2.21 GHz and 32 GB usable, 64-bit operating system using a parallel local machine with 8 processors.

### 2.3. Wall Shear Stress Parameters

The artery wall surface can be investigated using wall shear stress parameters in the x, y and z directions, the integral of which provides the time-averaged wall shear stress (TAWSS), as shown in Equation (4). Further artery wall parameters of interest are the oscillation shear index (OSI), given by Equation (5); the relative residence time (RRT) in Equation (6); and the endothelial cell activation potential (ECAP) provided in Equation (7).

(4) $$TAWSS=\frac{1}{T}\int_{0}^{T}\left|\overset{⃑}{WSS}\right|dt$$

(5) $$OSI=\frac{1}{2}\left(1-\frac{\left|\int_{0}^{T}\overset{⃑}{WSS}dt\right|}{TAWSS}\right)$$

(6) $$RRT=\frac{1}{(1-2\cdot OSI)\cdot TAWSS}$$

(7) $$ECAP=\frac{OSI}{TAWSS}$$

The WSS parameters in the three-dimensional analysis of the blood flow are essential to provide data on the endothelial cells’ behavior on the artery wall surface and are calculated using the OSI to assess the flow oscillation during a cardiac cycle (T). The RRT calculations for the same model with different mesh element sizes consider the oscillation of the stresses with its timed average. Finally, to assess the degree of thrombogenic susceptibility of the endothelial cells, the ECAP, a ratio between OSI and TAWSS, is calculated and compared amongst the different meshes. The artery wall parameters are solved using MATLAB (version R2023b) scripts to process the results for each simulation. Endothelial cell activation potential is an emerging hemodynamic parameter of significant clinical relevance, particularly in the context of aortic diseases, providing a comprehensive measure of the susceptibility of endothelial cells to activation and dysfunction under varying hemodynamic conditions. Its application in both research and clinical settings holds promise for improving the diagnosis, treatment, and prevention of aortic diseases, ultimately contributing to better cardiovascular outcomes [39].

### 2.4. Mesh Generation and Sensitivity

**Figure 3:**
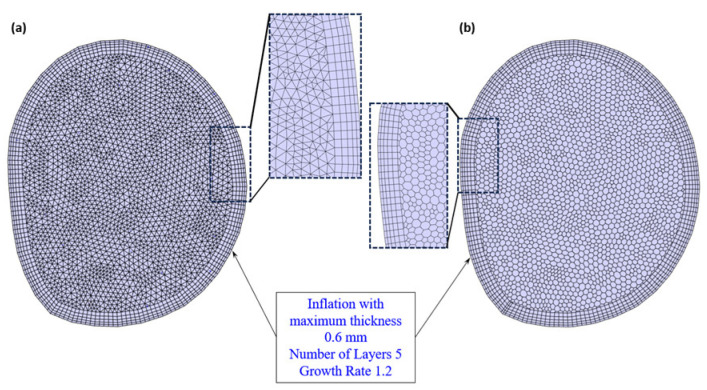
The mesh for the inlet face shows the inflation used for (**a**) tetrahedral and (**b**) polyhedral meshes.

In this study, the above arterial wall parameters were compared using two different mesh methods that are widely employed in the assessment of cardiovascular diseases: the tetrahedral mesh and the polyhedral mesh. These are shown in Figure 3. A mesh element size ranging from 0.2 mm to 1 mm, with a step size of 0.2, is used to generate five meshes for the tetrahedral model and five meshes for the polyhedral model. Comparisons are then made in terms of the following artery wall parameters, TAWSS, OSI, RRT, and ECAP, as well as the time taken for each simulation. The results of each model were evaluated in terms of accuracy and expedience of the simulation. The aim was to achieve clinically acceptable mesh results within a short timeframe. It was anticipated that a model with the capability to generate accurate results relatively quickly—in a non-invasive manner during a regular check-up—would be of assistance to medical doctors in assessing a patient’s risk of developing certain cardiovascular diseases that relate to endothelial cell damage. 

To achieve precise simulations of the velocity field near the blood surface wall, we considered the work of Soudah et al. [40] and Karimi et al. [41]. Soudah et al. [40] assessed an abdominal aortic aneurysm model computationally using a steady-state simulation to ensure the accuracy and validate the mesh sensitivity using a tetrahedral mesh with 2–2.5 million elements. Karimi et al.’s [41] mesh independence analysis was also performed using steady-state simulation at the average inlet velocity, and the grid was refined until independence was reached for the WSS with 1.77 million elements and tetrahedral and hexahedral meshes used. The boundary layer for the blood wall was set at 0.1 mm thickness, 5 layers [42], and a 1.2 growth rate of the thickness [2]. 

**Figure 4:**
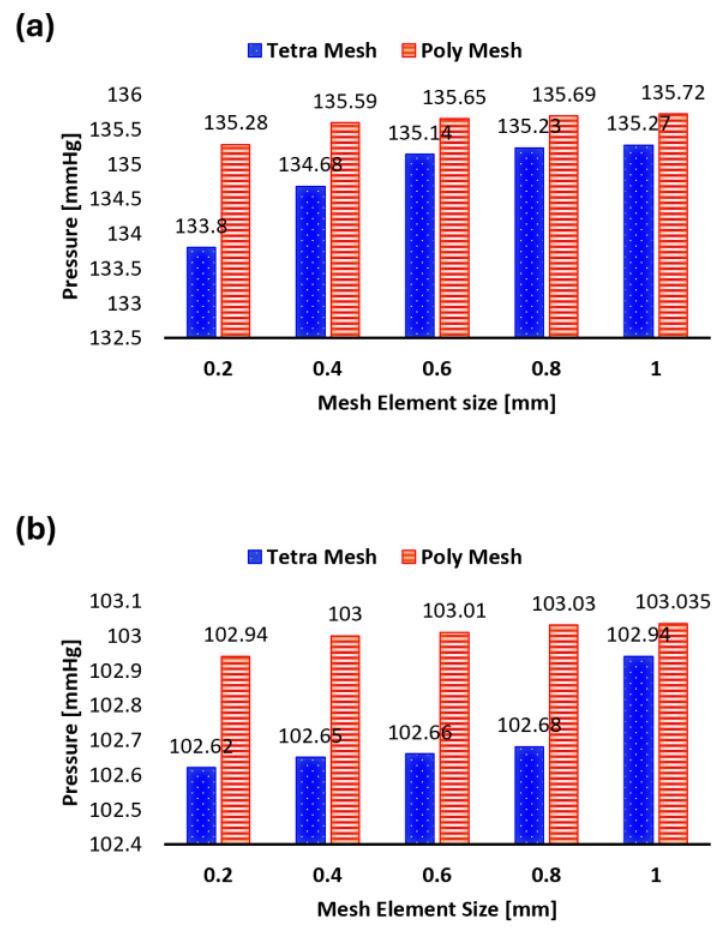
(**a**) The systolic pressure values for the different meshes; (**b**) the diastolic pressure values for the different meshes.

We performed the mesh independence test on the five tetrahedral meshes and the five polyhedral meshes. Figure 4a shows the systolic pressure values for both methods and Figure 4b shows the diastolic pressure values, with the simulation times on top of the bars. The test also compared the maximum WSS at the artery wall and the maximum velocity value at the outlet for the time period of 0.15 s (the peak of the inlet velocity used). Table 2 (A and B) shows the number of elements produced for each mesh for the healthy aorta as well the percentage error of the simulation for both the tetrahedral (A in Table 2) and polyhedral (B in Table 2) models. Notably, the error converges to 2% across all parameters of interest, with a 1% error achieved for the maximum velocity parameter with the polyhedral mesh. Overall, the 0.4 mm and 0.2 mm element sizes generated errors were within the acceptable range (2–3%) for simulations.

If we apply the most commonly used method for mesh sensitivity analysis—which consists of comparing the parameters of interest (V_max_, WSS_max_) at the systolic peak between each mesh—and we aim to use the least time-consuming method, as long as the difference between the two meshes does not exceed 3%, then, from A in Table 2, it can be seen that the tetrahedral mesh with a 0.4 mm element size is acceptable when V_max_ is the parameter of interest (a 50-min simulation time), whereas a 0.6 mm is acceptable when the WSS_max_ is the parameter of interest (a 35-min simulation time). However, for the polyhedral meshes, 0.6 mm would be acceptable if we use Vmax as the parameter of interest (a 31-min simulation time), whilst only 0.2 mm is acceptable if WSS_max_ is the parameter of interest (a 103-min simulation time), as shown in B in Table 2.

## 3. Results

In this study, we performed five meshes using tetrahedral elements and five meshes using polyhedral elements for a healthy subject to identify which meshes generate the greatest accuracy and how long the simulations take under the different models. In this section, the model validation is presented and the pressure waveforms are presented—to find the pressure difference between the tetrahedral and polyhedral methods and the endothelial cell analysis to the arterial wall surface. A previous study conducted by He et al. [43] highlighted the importance of mesh type in cardiovascular simulations, emphasizing the balance between accuracy and computational efficiency. Image-based simulation of vascular biomechanics is a rapidly evolving research area, with significant implications for understanding the initiation, progression, and treatment of vascular diseases [43]. This study builds on those insights, aiming to identify the optimal mesh configuration that maximizes accuracy while managing computational demands.

In our study, we obtain an average velocity of 0.14 m/s and an equivalent diameter of 35.88 mm, with a Reynolds Number (Re) of around 1331, which is a laminar regime. This is consistent with findings from the similar literature that make use of clinical data to generate boundary conditions. For example, Tse et al. [44] used clinical data and showed that the 0.2 mm mesh element size could generate a number of elements from 500,000 to 900,000, which is acceptable for aortic geometry for transient analysis with the assumption that the blood flow is laminar based on the Reynolds number (Re) not exceeding 2389 for their pulsatile flow. Their aortic model was solved as incompressible, homogeneous, and with Newtonian blood properties, and the aorta wall was rigid with a no-slip boundary condition.

### 3.1. Validation of the Study

**Figure 5:**
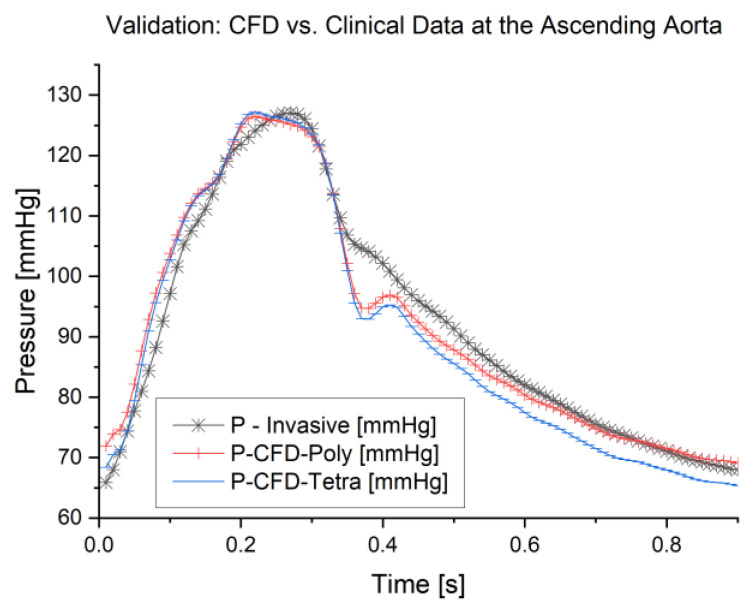
The computational fluid dynamics result was validated against the clinical data for the mesh element size of 0.2 mm for both polyhedral and tetrahedral meshes.

The results of these simulations were validated against the clinical data obtained. Since we imposed blood flow as an inlet boundary condition at the ascending aorta, we compared our results against the pressure waveform obtained clinically and the results from the CFD simulation. The validation is based on the aortic model with a mesh element size of 0.2 mm for tetrahedral and polyhedral meshes. The validation outcome, as shown in Figure 5, illustrates the pressure waveforms for the CFD simulations generated by ANSYS Fluent for both meshes (tetrahedral and polyhedral) on the same track for the clinical pressure waveform at the ascending aorta. For further analysis, we found that the average error for the waveforms for the 0.2 mm element size is 3.3% for the polyhedral mesh and 3.1% for the tetrahedral mesh, whilst the error between both meshes is 0.5%. Furthermore, the CFD modeling for the 0.2 mm polyhedral mesh took 103 min; however, for the tetrahedral mesh, this took 222 min. Therefore, we can achieve a 54% reduction in simulation time with a negligible difference between the models. The validation outcome is within the acceptable range and supports the findings of the previous literature, such as Elti et al. [45] and Leuprecht et al. [46], which show that the CFD results could be validated with the boundary conditions obtained clinically using MRI. Therefore, this method presents a promising tool that could be used for the early diagnosis of any abnormalities in the aorta.

### 3.2. Pressure Waveform

**Figure 6:**
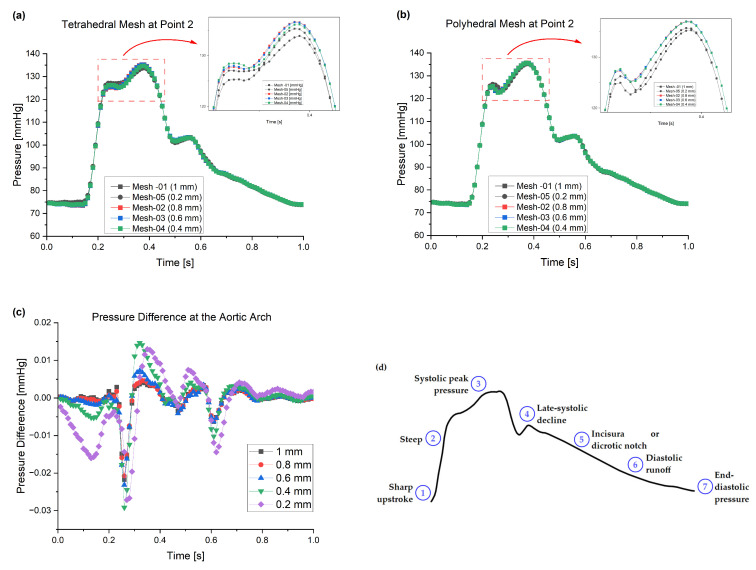
The pressure waveforms at the aortic arch for the different meshes: (**a**) tetrahedral; (**b**) polyhedral; (**c**) the ΔP2 at the aortic arch, showing that the difference between the meshes is minimal; and (**d**) the normal central aortic pressure waveform.

The normal central aortic waveform is composed of seven distinct components: (1) the initial systolic upstroke; (2) a steep, uninterrupted rise in the incident wave; (3) the systolic peak pressure; (4) a late-systolic decline accompanied by a deceleration in forward blood flow; (5) the incisura or dicrotic notch, which marks the closure of the aortic valve; (6) the diastolic runoff; and (7) the end-diastolic pressure (as illustrated in Figure 6d). The shape and contour of the aortic waveform are influenced by several factors, including the duration of systole, mean arterial pressure, vasomotor tone, pulse wave velocity, and wave reflections [43].

For these meshes, we conducted a deeper examination of the pressure waveforms at the aortic arch (the location of the points shown in Figure 2b) to see the difference incurred by reducing the element size for each mesh method. Figure 6 shows the pressure waveform for the element sizes between 0.2 mm and 1 mm in both the tetrahedral and polyhedral meshes.

The pressure waveforms at the aortic arch show that reducing the mesh element size does not change the results materially; for example, the average pressure difference between the 0.4 mm and 1 mm tetrahedral meshes is 0.28 mmHg compared to polyhedral mesh at 0.06 mmHg. However, the average difference between the 0.2 and 1 mm meshes is 0.36 and 0.27 mmHg for the tetrahedral and polyhedral types, respectively. Another comparison between the same mesh element size of 0.4 mm and 0.2 mm for both meshes yields 0.41 and 0.61 mmHg, respectively. Also, both meshes produce the same shape of the pressure waveform at the aortic arch, which validates our approach and justifies acceptance of the 0.4 mm polyhedral mesh, the simulation that took only 37 min.

Figure 6c shows the essential values for the pressure difference for these meshes, which confirms the method is robust. Furthermore, the short simulation times whilst achieving suitable CFD results indicate that medical practitioners could make use of these models to provide timely information without requiring specialized computational equipment. The results for this particular patient (having aortic dimensions as shown in Table 1) suggest using a 0.4 mm element size for tetrahedral or polyhedral meshes could obtain the characteristics of the artery wall within 37–50 min. This type of simulation could allow for more non-invasive data to be rendered for a patient during an annual check-up. This could be especially useful for demographic groups that have constrained access to tertiary healthcare (such as rural populations), or who have a higher risk of developing cardiovascular disease.

### 3.3. Endothelial Cell Characteristics

**Figure 7:**
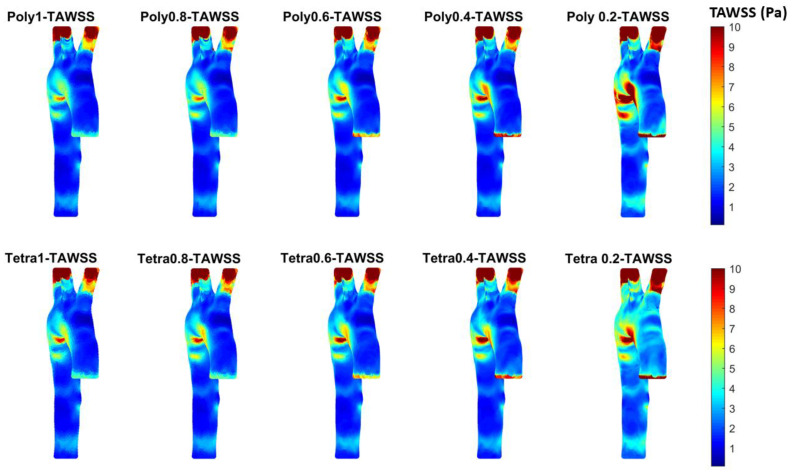
TAWSS for the different meshes to see the development of high contours for healthy aorta. (Tetrahedral = Tetra; polyhedral = Poly).

The pressure waveforms provide essential data but not enough information about the artery wall. Therefore, in this study, we investigated many hemodynamical characteristics that are calculated based on the WSS in the x, y and z directions using ANSYS Fluent for the different meshes. The WSS data were processed in MATLAB to calculate the TAWSS, as shown in Figure 7, to allocate the high contours for TAWSS. The time average for the shear stress contours was investigated with the assumption of laminar flow in a healthy aorta that impacts the endothelial cells as the blood exerts mechanical forces on the aortic wall [47,48,49]. Unhealthy arteries attain high values of TAWSS in cases of atherosclerosis or arterial blockages, whereas low TAWSS (<0.4 Pa) stimulates the proatherogenic endothelial phenotype [50]. Also, high values of TAWSS are accompanied by a low oscillatory shear index (OSI).

The TAWSS figure shows a similar pattern in the different meshes, characterized by high and low values in the same regions; however, if we take a closer look to compare between the two extreme meshes (i.e., 0.2 and 1 mm), it can be seen that the 0.2 mm meshes show a larger surface with high TAWSS compared to the 1 mm meshes for both tetrahedral and polyhedral types. Even if we compare between the selected meshes with the most commonly used method for mesh sensitivity (i.e., 0.6 mm and 0.4 mm for tetrahedral and polyhedral meshes, respectively) and the finest meshes (i.e., 0.2 mm) from Table 2 (A and B), it can be seen that for the tetrahedral mesh, 0.4 mm is acceptable if we consider Vmax and/or WSSmax as the parameters of interest; however, for the polyhedral mesh, 0.4 mm could be acceptable if we use Vmax as the parameter of interest, or 0.2 mm if the WSSmax is the parameter of interest and if both parameters need to be considered (i.e., Vmax and WSSmax).

**Figure 8:**
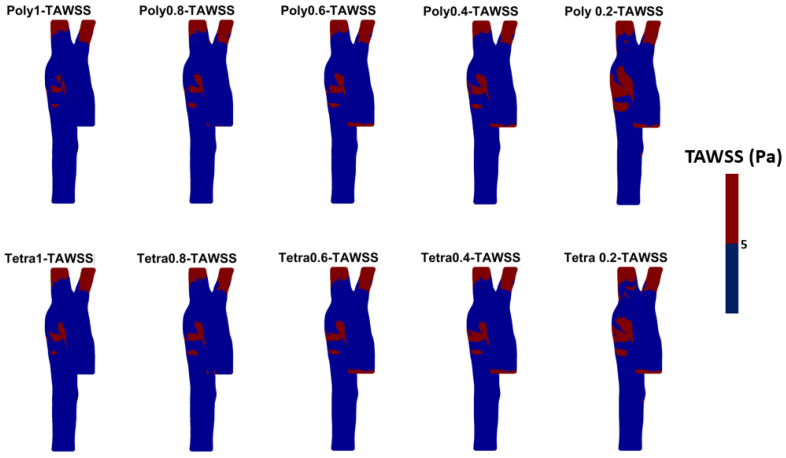
The threshold values for the TAWSS for the different meshes (blue for values below the threshold of 5 Pa and red for values above 5 Pa) are used to visualize areas higher and lower than the threshold value. (Tetra = tetrahedral; Poly = polyhedral).

**Figure A5:**
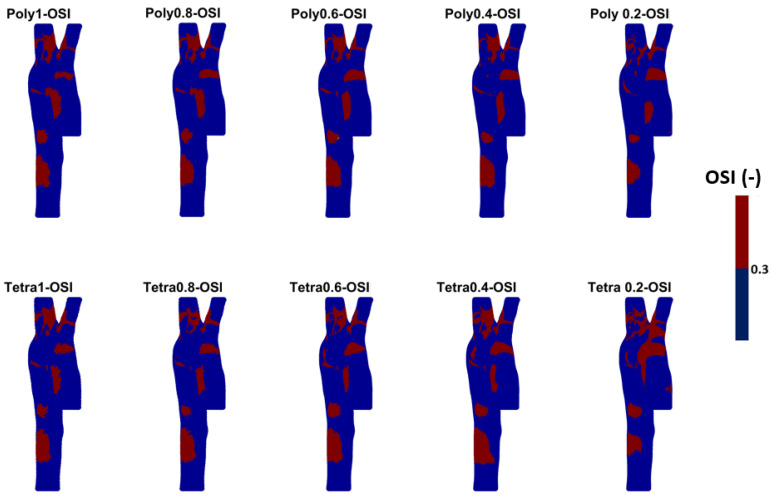
The threshold values of OSI for the different meshes presented in this study illustrate the high and low mechanical shear oscillation WSS per cardiac cycle on the aortic wall. (Tetrahedral = Tetra; polyhedral = Poly).

**Figure A6:**
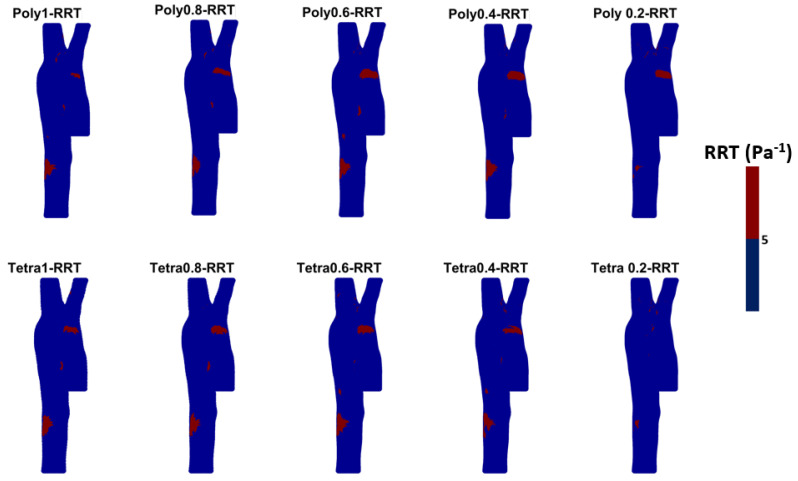
The threshold contours for the RRT for the different meshes illustrate the relative residence time based on the mechanical oscillation during a cardiac cycle and WSS values on the aortic wall. (Tetrahedral = Tetra; polyhedral = Poly).

**Figure A7:**
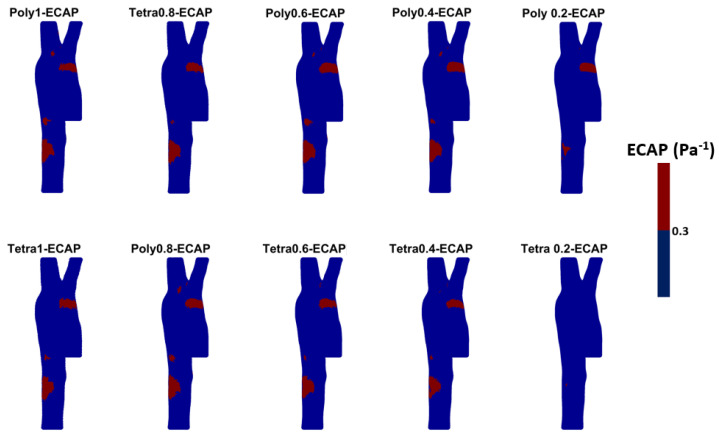
The threshold for the ECAP for the different meshes illustrates the degree of thrombogenic susceptibility of the endothelial cell to localize the possible location for high mechanical oscillation. (Tetrahedral = Tetra; polyhedral = Poly).

The WSS-derived parameters (i.e., TAWSS, OSI, RRT, ECAP, and others) are widely used in CFD studies related to both healthy and diseased aortic models. From the literature, we can find that for each parameter a threshold value is considered [51,52,53,54,55]. Figure 8 shows the TAWSS with a threshold value (in diseased patients 0.2 to 0.3 Pas is usually used); here, as a demonstration, we have used 5 Pa, and from these results it can be seen that by increasing mesh size, the area of TAWSS of >5 Pa increases significantly. Similarly, from the OSI, RRT, and ECAP threshold figures (see, respectively, Figure A5, Figure A6 and Figure A7 in Appendix B) it could be observed that mesh size has an effect on the areas of low or high WSS-derived parameters. This could be accentuated in diseased cases where the artery is curved, inflated (aneurysms), or deflated (stenosis).

**Figure 9:**
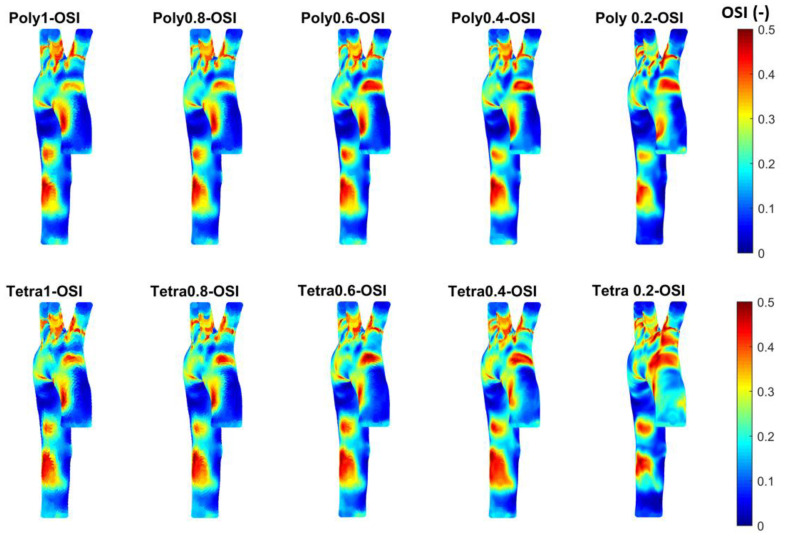
The OSI for the different meshes to see the development of high contours for healthy aorta. (Tetrahedral = Tetra; polyhedral = Poly).

The OSI was also calculated in MATLAB using Equation (5), with a focus on both the range of 0–0.5 to see the high contours for the OSI and on which mesh is suitable with the time taken to achieve the results, as shown in Figure 9. The OSI contours were used to define the oscillation of the blood flow and to determine the zones on the artery wall where high OSI could be used to assess the endothelial dysfunction and atherogenesis. Figure 9 shows that the reduction in mesh element size, using polyhedral or tetrahedral mesh methods, could indicate an endothelial dysfunction at the ascending aorta, bifurcation, and before the abdominal aorta in terms of OSI.

**Figure 10:**
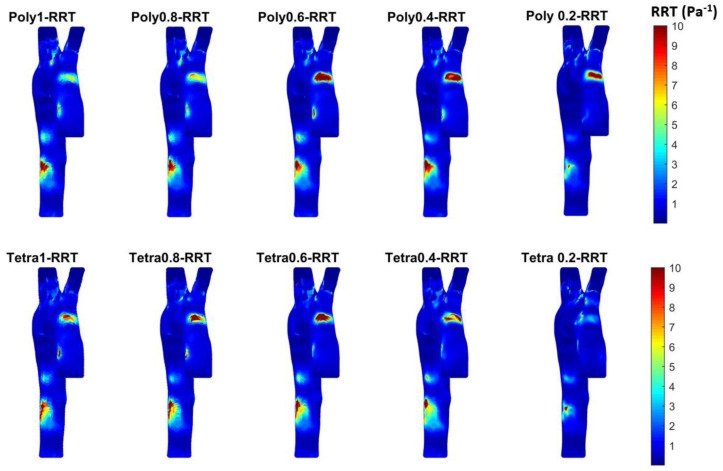
The RRT for the different meshes to see the development of high contours for healthy aorta. (Tetrahedral = Tetra; polyhedral = Poly).

To assess the TAWSS and OSI impact, we calculated the relative residence time (RRT) (${Pa}^{-1}$) to produce contours, as shown in Figure 10. RRT was introduced by Himburg et al. [56] to study the effect of the residence time of plasma and blood particles on the atherosclerotic process. Notably, RRT is calculated based on the time-averaged WSS and OSI. Therefore, RRT is defined as the inverse of the time-averaged WSS vector marginated, which is a qualitative factor. The WSS vector can quantify the near-wall fluid velocity and can measure RRT by the Eulerian model. The RRT is proportional to the residence time of the blood particles near the artery wall calculated using Equation (6). RRT is a dimensional index used to illustrate the contours of low and oscillating shear developed by the blood flow based on the WSS [56,57,58,59]. Recent studies [12,34,44,50,60,61] used Newtonian and non-Newtonian blood properties for simulating arteries using CFD techniques. These studies indicated that in arteries with changes to the cross-sectional blood flow due to the structure—such as bifurcation and branching or arterial blockages due to stenosis—the Newtonian blood properties do not mask the shear-thinning characteristic of the blood. Additionally, constant viscosity for the blood does not address correctly the flow circulation and reverse flow in complicated aortic geometry. Kumar et al. [34] found that the Newtonian properties for large arteries show that low WSS regions over-exaggerate the hemodynamic parameters during the narrowing of the artery. Tse et al. [44] conform that the aorta—as it is always composed of irregular contours, numerous bends, bulging supra-aortic branches, and a large degree of curvature at the arch—requires the use of non-Newtonian blood properties for the prediction of any abnormalities. Therefore, using the Carreau model provides more accurate TAWSS contours at the regions where instantaneous WSS deviates from the main flow direction for a large fraction of the cardiac cycle. In our study, the healthy aortic geometry with a non-Newtonian (Carreau) model displays high contours close to the ascending and aortic arch and close to the abdominal aorta. These contours become clearer with the polyhedral mesh as the shape of the polyhedral cells provides a better gradient and approximation to the neighboring cells. Also, the polyhedral mesh is less sensitive to stretching compared to the tetrahedral method, which provides improved results for the stability of the CFD model [56,57,58,59]. The 0.4 mm element size shows the RRT contours could impact the endothelia at the top of the ascending aorta close to the aortic arch or close to the abdominal aortic area.

There is a clear correlation between the OSI and TAWSS as established by the ratio of these two values, called the endothelial cell activation potential (ECAP), which quantifies the degree of thrombogenic susceptibility of the aorta wall. High concentrations of contours for the ECAP correspond to contours of high OSI and low TAWSS as an indication of a region with susceptibility to high endothelial cell deposition and thrombosis [58].

**Figure 11:**
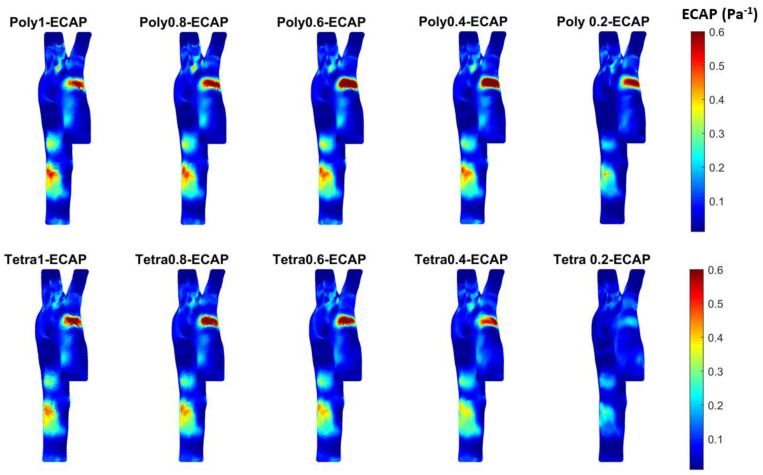
The ECAP for the different meshes to see the development of high contours for healthy aorta. (Tetrahedral = Tetra; polyhedral = Poly).

Figure 11 shows the ECAP for the different meshes calculated in MATLAB based on the WSS in three dimensions obtained from ANSYS Fluent. The results of the 0.2 mm polyhedral mesh show high contours of the ECAP at the areas of low TAWSS, which is the opposite for the case with abdominal aortic aneurysm [51,58,59].

## 4. Discussion

The main findings of this study include the following:

1. Both tetrahedral and polyhedral meshes at a 0.2 mm element size provided high accuracy, with validation against clinical data showing an average error of 3.3% for polyhedral and 3.1% for tetrahedral meshes. Notably, the polyhedral mesh achieved a 54% reduction in simulation time (103 min) compared to the tetrahedral mesh (222 min) while maintaining comparable results;
2. The pressure waveform analysis indicated minimal differences between mesh types and sizes. A 0.4 mm polyhedral mesh was found to be a practical balance, producing accurate waveforms in just 37 min;
3. WSS-based parameters (TAWSS, OSI, RRT, and ECAP) revealed similar trends across both mesh types, though finer meshes captured more detailed variations. The polyhedral mesh demonstrated better gradient handling, making it suitable for simulating complex vascular regions like the aortic arch.

These findings suggest that a polyhedral mesh with a 0.4 mm element size is optimal for routine clinical applications, providing reliable hemodynamic data swiftly and supporting the use of CFD models for non-invasive cardiovascular diagnostics, especially in resource-limited settings.

The findings of this study underscore the significance of mesh selection in CFD simulations for accurately capturing hemodynamic parameters, particularly wall shear stress (WSS) derivatives like TAWSS, OSI, RRT, and ECAP. These parameters are essential for understanding the biomechanical forces acting on arterial walls, providing crucial insights into both healthy and pathological aortic conditions [2].

Our results show that the polyhedral mesh, particularly with a 0.4 mm element size, offers a balanced approach by delivering high accuracy while significantly reducing computational time, as evidenced by the 54% reduction compared to tetrahedral meshes. The pressure waveform analysis demonstrated negligible differences between tetrahedral and polyhedral meshes, validating the use of a 0.4 mm polyhedral mesh for clinical purposes, with the entire simulation taking only 37 min.

The analysis of WSS derivatives reveals that finer meshes are critical for detecting regions with disturbed flow, particularly in curved or bifurcated segments of the aorta where lower TAWSS values and higher OSI levels are more likely to occur. These areas are known for promoting endothelial dysfunction, inflammation, and atherosclerosis development. This is due to the reduced shear stress, which promotes endothelial cell dysfunction, lipid accumulation, and inflammation. The observed increase in the area of TAWSS > 5 Pa with larger mesh sizes indicates a greater sensitivity of the model in detecting regions with potentially protective high shear stress, emphasizing the importance of mesh resolution in accurately capturing these critical areas [59]. The impact of mesh size on OSI, as shown in the results, suggests that finer meshes may be necessary to accurately capture these oscillatory flow regions, particularly in diseased arteries where geometry alterations such as curvature, dilation (aneurysms), or narrowing (stenosis) can exacerbate flow disturbances [61,62]. Our results align with previous studies, indicating that mesh size directly affects the sensitivity of detecting such hemodynamic abnormalities.

Further, this study highlights that in regions prone to pathological conditions, such as aneurysms or stenosis, accurately modeling RRT and ECAP is paramount for assessing thrombotic risks and disease progression. The findings suggest that higher-resolution meshes better capture complex flow patterns, allowing for more precise predictions of disease outcomes. These insights advocate for the integration of CFD simulations in routine clinical diagnostics, enabling early detection and more tailored interventions for patients at risk of cardiovascular diseases, particularly in settings with limited resources or specialized equipment [63]. The sensitivity of ECAP to mesh size underscores the need for precise modeling for identifying regions at high risk for endothelial activation, especially in aortic disease, where altered flow patterns can significantly impact EC function [61].

In diseased aortic models, such as those with aneurysms or stenosis, accurately modeling wall shear stress derivatives becomes critical due to the complex flow patterns that arise from altered geometries. In aneurysmal regions, low TAWSS and high OSI are commonly observed, conditions that promote further dilation and potential rupture. Similarly, stenotic arteries exhibit high shear stress upstream and low shear stress downstream of the stenosis, creating an environment that encourages plaque progression and thrombosis [64]. The results of this study indicate that mesh size has a significant impact on capturing these subtle hemodynamic changes, with higher-resolution meshes proving essential for reliable predictions of disease progression. This highlights the importance of precise mesh configuration when simulating pathological conditions, ensuring that CFD models can accurately inform clinical decisions and improve patient outcomes.

These findings highlight the crucial role of WSS derivatives in cardiovascular disease modeling and underscore the importance of mesh resolution in achieving accurate hemodynamic assessments. Enhancing computational models to better capture these parameters enables clinicians and researchers to more effectively predict and manage the risks associated with aortic pathologies [64].

### Limitations and Further Study

This study contains some limitations that will be addressed in our future work. The current CFD results, which show a promising technique to enable relatively quick simulation of hemodynamic characteristics of the artery, are based on a single healthy patient model. The rigid wall assumption is the current method used for this type of simulation; however, future work to enhance this study will include using a deformable arterial wall with the fluid–structure interaction (FSI) method [65,66] with the polyhedral mesh to reduce simulation time. The anticipated outcome of the FSI is to address the changes to the WSS resulting from the transient structural analysis, which will be used to calculate the TAWSS. These TAWSS contours will illustrate the backflow of the propagated pressure waveforms, which will result in reducing the high contours of TAWSS compared to the rigid wall at the aortic arch as it will generate smoother TAWSS contours therein.

Future work would expand the examination to several healthy patients and investigate the effects of different cardiovascular diseases on the output. Furthermore, the current approach applies to the aorta but cannot necessarily be applied to smaller arteries. Additionally, future work could include considering the body’s acceleration and thermal effects for different blood rheological properties, such as the Casson model.

## 5. Conclusions

This study demonstrates that accurate CFD results for aortic assessments can be achieved in a relatively short time using accessible computing resources. For routine evaluations, a 0.4 mm element size for either tetrahedral or polyhedral meshes offers a practical balance between speed and accuracy. If maximum blood velocity is the focus, a 0.2 mm polyhedral mesh is recommended, while a 0.4–0.6 mm tetrahedral mesh is better-suited for analyzing maximum wall shear stress. These simulations provide a valuable, non-invasive tool for the early detection and monitoring of aortic abnormalities, especially for populations with limited access to specialized healthcare. Additionally, incorporating WSS derivatives in sensitivity analyses can guide better clinical decisions and improve aortic disease management.

## Figures and Tables

**Table 1:** The aortic geometry dimensions and boundary conditions used, including blood flow and pressure waveforms (adapted from [2] under the Creative Commons Attribution 4.0 International license).

Location	Diameter (mm)	Area (mm^2^)	Boundary Condition	Waveform
Ascending aorta (AA)	35.88	100.15	Ascending inlet blood velocity waveform	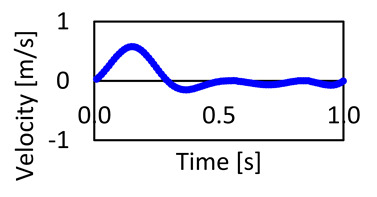
Iliac (I)	29.74	65.58	Iliac outlet blood pressure waveform	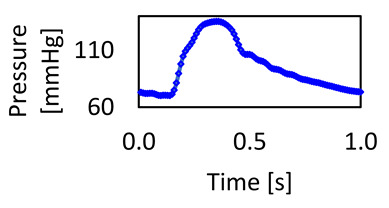
Brachiocephalic (BC)	13.48	14.02	Outlet blood pressure waveform	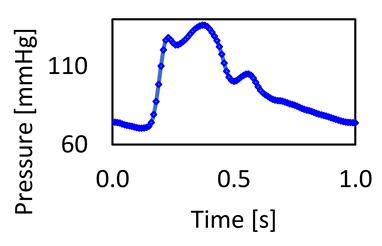
Left common carotid (LCC)	8.43	5.36		
Left subclavian (LS)	11.75	10.00		

**Table 2:** (A) The tetrahedral mesh independence test for the maximum velocity at the outlet and maximum wall shear stress (WSS) at the artery wall during the period of 0.15 s (the peak of the blood flow). (B) The polyhedral mesh independence test for the maximum velocity at the outlet and maximum wall shear stress (WSS) at the artery wall during the period of 0.15 s (the peak of the blood flow).

**(A)**						
**Element Size [mm]**	**Element Number (T)**	**V_max_(T) [m/s]**	**%**	**WSS_max_(T) [Pa]**	**%**	**Time (T) (min)**
0.2	7,030,641	0.7200	2%	21.8233293	2%	222
0.4	1,148,142	0.7075	3%	22.3736838	3%	50
0.6	547,059	0.6854	6%	23.0381622	3%	35
0.8	405,490	0.6442	13%	23.6513115	12%	31
1	369,728	0.5607		26.7444558		30
**(B)**						
**Element Size [mm]**	**Element Number (P)**	**V_max_(P) [m/s]**	**%**	**WSS_max_(P) [Pa]**	**%**	**Time (P) (min)**
0.2	9,248,899	0.753678	1%	19.67837	2%	103
0.4	1,753,454	0.748364	1%	20.14034	6%	37
0.6	951,802	0.741864	2%	21.35883	6%	31
0.8	766,309	0.725355	4%	22.67753	8%	27
1	732,146	0.753678		24.66996		25

(T) = Tetrahedral mesh method. (P) = Polyhedral mesh method.

**Figure A4:**
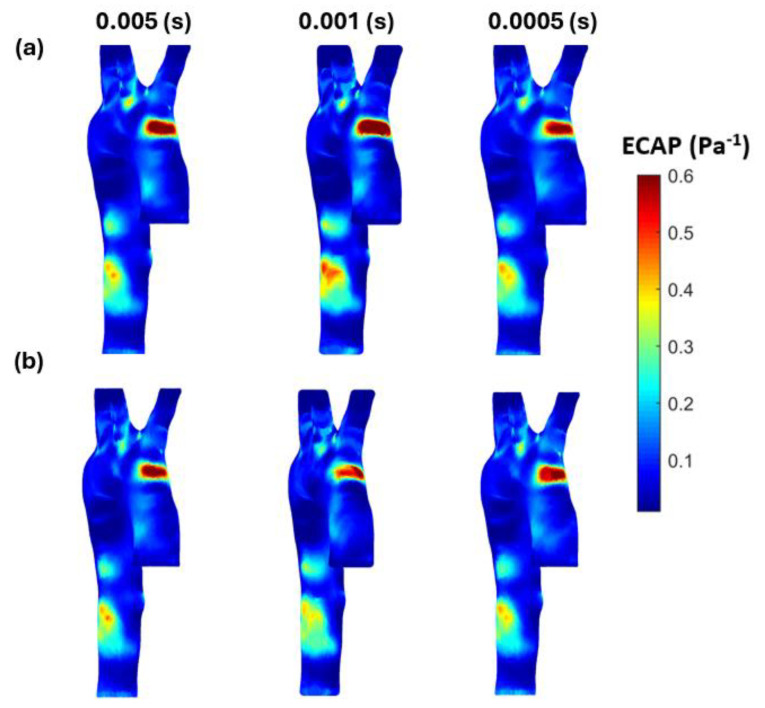
The ECAP for the three-time steps (0.005, 0.001, and 0.0005 s) for 0.4 mm mesh element size: (**a**) polyhedral mesh; and (**b**) tetrahedral mesh.

## Data Availability

The original contributions presented in the study are included in the article, further inquiries can be directed to the corresponding author/s.

## References

[1] Martin S.S., Aday A.W., Almarzooq Z.I., Anderson C.A., Arora P., Avery C.L., Baker-Smith C.M., Gibbs B.B., Beaton A.Z., Boehme A.K. (2024). 2024 Heart Disease and Stroke Statistics: A Report of US and Global Data From the American Heart Association. Circulation.

[2] AL-Rawi M., AL-Jumaily A.M., Belkacemi D. (2022). Non-invasive diagnostics of blockage growth in the descending aorta-computational approach. Med. Biol. Eng. comput..

[3] Conrad N., Molenberghs G., Verbeke G., Zaccardi F., Lawson C., Friday J.M., Su H., Jhund P.S., Sattar N., Rahimi K. (2024). Trends in cardiovascular disease incidence among 22 million people in the UK over 20 years: Population-based study. BMJ.

[4] Timmis A., Vardas P., Townsend N., Torbica A., Katus H., de Smedt D., Gale C.P., Maggioni A.P., Petersen S.E., Huculeci R. (2022). European Society of Cardiology. European Society of Cardiology: Cardiovascular disease statistics 2021. Eur. Heart J..

[5] Andersson C., Vasan R.S. (2018). Epidemiology of cardiovascular disease in young individuals. Nat. Rev. Cardiol..

[6] Abdalla S.M., Yu S., Galea S. (2020). Trends in Cardiovascular Disease Prevalence by Income Level in the United States. JAMA Netw. Open.

[7] Roth G.A., Mensah G.A., Johnson C.O., Addolorato G., Ammirati E., Baddour L.M., Barengo N.C., Beaton A.Z., Benjamin E.J., Benziger C.P. (2020). GBD-NHLBI-JACC Global Burden of Cardiovascular Diseases Writing Group. Global Burden of Cardiovascular Diseases and Risk Factors, 1990–2019: Update From the GBD 2019 Study. J. Am. Coll. Cardiol..

[8] National Center for Health Statistics Multiple Cause of Death 2018–2021 on CDC WONDER Database. https://wonder.cdc.gov/mcd.html.

[9] Tsao C.W., Aday A.W., Almarzooq Z.I., Anderson C.A., Arora P., Avery C.L., Baker-Smith C.M., Beaton A.Z., Boehme A.K., Buxton A.E. (2023). Heart Disease and Stroke Statistics—2023 Update: A Report From the American Heart Association. Circulation.

[10] Healthdirect Australia Heart Foundation. Health Direct. https://www.healthdirect.gov.au/partners/heart-foundation.

[11] Heart Foundation, New Zealand Latest Heart Disease Statistics. https://www.heartfoundation.org.nz/statistics.

[12] Al-Rawi M., Al-Jumaily A.M. (2016). Assessing abdominal aorta narrowing using computational fluid dynamics. Med. Biol. Eng. Comput..

[13] Zhu Y. (2018). Clinical validation and assessment of aortic hemodynamics using computational fluid dynamics simulations from computed tomography angiography. BioMed. Eng. Online.

[14] Numata S., Itatani K., Kanda K., Doi K., Yamazaki S., Morimoto K., Manabe K., Ikemoto K., Yaku H. (2016). Blood flow analysis of the aortic arch using computational fluid dynamics. Eur. J. Cardio-Thorac. Surg..

[15] Al-Rawi M., Al-Jumaily A., Belkacemi D. (2022). Do Long Aorta Branches Impact on the Rheological Properties?. Proceedings of the ASME 2021 International Mechanical Engineering Congress and Exposition.

[16] Kamrath B.D., Suess T.N., Gent S.P. (2016). Assessment of Pulsatile Blood Flow Models for the Descending Aorta Using CFD. Proceedings of the ASME 2015 International Mechanical Engineering Congress and Exposition.

[17] Gao F., Ohta O., Matsuzawa T. (2008). Fluid-structure interaction in layered aortic arch aneurysm model: Assessing the combined influence of arch aneurysm and wall stiffness. Australas. Phys. Eng. Sci. Med..

[18] Algabri Y.A., Rookkapan S., Gramigna V., Espino D.M., Chatpun S. (2019). Computational study on hemodynamic changes in patient-specific proximal neck angulation of abdominal aortic aneurysm with time-varying velocity. Australas. Phys. Eng. Sci. Med..

[19] Alkhatib F., Wittek A., Zwick B.F., Bourantas G.C., Miller K. (2023). Computation for biomechanical analysis of aortic aneurysms: The importance of computational grid. Comput. Methods Biomech. Biomed. Eng..

[20] Takizawa K., Tezduyar T.E., Uchikawa H., Terahara T., Sasaki T., Yoshida A. (2019). Mesh refinement influence and cardiac-cycle flow periodicity in aorta flow analysis with isogeometric discretization. Comput. Fluids.

[21] Zhu C., Seo J., Mittal R. (2018). Computational modelling and analysis of haemodynamics in a simple model of aortic stenosis. J. Fluid. Mech..

[22] Trenti C., Ziegler M., Bjarnegã¥Rd N., Ebbers T., Lindenberger M., Dyverfeldt P. (2022). Wall shear stress and relative residence time as potential risk factors for abdominal aortic aneurysms in males: A 4D flow cardiovascular magnetic resonance case–control study. J. Cardiovasc. Magn. Reason..

[23] Tang X., Wu C. (2024). A predictive surrogate model for hemodynamics and structural prediction in abdominal aorta for different physiological conditions. Comput. Methods Programs Biomed..

[24] Simão M., Ferreira J., Tomás A.C., Fragata J., Ramos H.M. (2017). Aorta Ascending Aneurysm Analysis Using CFD Models towards Possible Anomalies. Fluids.

[25] Petuchova A., Maknickas A. (2021). Computational analysis of aortic haemodynamics in the presence of ascending aortic aneurysm. Technol. Health Care.

[26] Minderhoud S.C., Arrouby A., Hoven A.T.v.D., Bons L.R., Chelu R.G., Kardys I., Rizopoulos D., Korteland S.-A., Bosch A.E.v.D., Budde R.P. (2024). Regional Aortic Wall Shear Stress Increases over Time in Patients with a Bicuspid Aortic Valve. J. Cardiovasc. Magn. Reson..

[27] Minderhoud S.C.S., Roos-Hesselink J.W., Chelu R.G., Bons L.R., Hoven A.T.v.D., Korteland S.-A., Bosch A.E.v.D., Budde R.P.J., Wentzel J.J., Hirsch A. (2022). Wall shear stress angle is associated with aortic growth in bicuspid aortic valve patients. Eur. Heart J. Cardiovasc. Imaging.

[28] Soulat G., Scott M.B., Allen B.D., Avery R., Bonow R.O., Malaisrie S.C., McCarthy P., Fedak P.W., Barker A.J., Markl M. (2022). Association of Regional Wall Shear Stress and Progressive Ascending Aorta Dilation in Bicuspid Aortic Valve. JACC Cardiovasc. Imaging.

[29] Chiu J.J., Chien S. (2011). Effects of disturbed flow on vascular endothelium: Pathophysiological basis and clinical perspectives. Physiol. Rev..

[30] Osswald A., Karmonik C., Anderson J., Rengier F., Kretzler M., Engelke J., Kallenbach K., Kotelis D., Partovi S., Böckler D. (2017). Elevated Wall Shear Stress in Aortic Type B Dissection May Relate to Retrograde Aortic Type A Dissection: A Computational Fluid Dynamics Pilot Study. Eur. J. Cardiothorac. Surg..

[31] Meng X., Wu J., Chen D., Wang C., Wu Y., Sun T., Chen J. (2023). Ascending aortic volume: A feasible indicator for ascending aortic aneurysm elective surgery?. Acta Biomater..

[32] Qu W., Li X., Huang H., Xie C., Song H. (2022). Mechanisms of the ascites volume differences between patients receiving a left or right hemi-liver graft liver transplantation: From biofluidic analysis. Comput. Methods Programs Biomed..

[33] Caballero A., Laín S. (2013). A review on Computational fluid dynamics modelling in human thoracic aorta. Cardiovasc. Eng. Tech..

[34] Kumar N., Khader A., Pai R., Kyriacou P., Khan S., Koteshwara P. (2019). Computational fluid dynamic study on effect of Carreau-Yasuda and Newtonian blood viscosity models on hemodynamic parameters. J. Comput. Methods Sci. Eng..

[35] Spiegel M., Redel T., Zhang Y., Struffert T., Hornegger J., Grossman R., Doerfler A., Karmonik C. Tetrahedral and polyhedral mesh evaluation for cerebral hemodynamic simulation-A comparison. Proceedings of the 2009 Annual International Conference of the IEEE Engineering in Medicine and Biology Society.

[36] Wellnhofer E., Goubergrits L., Kertzscher U., Affeld K., Fleck E. (2009). Novel non-dimensional approach to comparison of wall shear stress distributions in coronary arteries of different groups of patients. Atherosclerosis.

[37] Martelli F., Milani M., Montorsi L., Ligabue G., Torricelli P. (2019). Fluid-Structure Interaction of Blood Flow in Human Aorta Under Dynamic Conditions: A Numerical Approach. Proceedings of the ASME 2018 International Mechanical Engineering Congress and Exposition.

[38] Al-Rawi M., Belkacemi D., Lim E.T.A., Khashram M. (2024). Investigation of Type A Aortic Dissection Using Computational Modelling. Biomedicines.

[39] Wang X., Shen Y., Shang M., Liu X., Munn L.L. (2023). Endothelial mechanobiology in atherosclerosis. Cardiovasc. Res..

[40] Soudah E., Ng E.Y.K., Loong T.H., Bordone M., Pua U., Narayanan S. (2013). CFD Modelling of Abdominal Aortic Aneurysm on Hemodynamic Loads Using a Realistic Geometry with CT. Comput. Math. Methods Med..

[41] Karimi S., Dabagh M., Vasava P., Dadvar M., Dabir B., Jalali P. (2014). Effect of rheological models on the hemodynamics within human aorta: CFD study on CT image-based geometry. J. Non-Newton. Fluid Mech..

[42] Cilla M., Casales M., Peña E., Martínez M.Á., Malvè M. (2020). A parametric model for studying the aorta hemodynamics by means of the computational fluid dynamics. J. Biomech..

[43] He Y., Northrup H., Le H., Cheung A.K., Berceli S.A., Shiu Y.T. (2022). Medical Image-Based Computational Fluid Dynamics and Fluid-Structure Interaction Analysis in Vascular Diseases. Front. Bioeng. Biotechnol..

[44] Tse K.M., Chang R.S., Lee H.P., Lim S.P., Venkatesh S.K., Ho P. (2012). A computational fluid dynamics study on geometrical influence of the aorta on haemodynamics. Eur. J. Cardiothorac. Surg..

[45] Etli M., Canbolat G., Karahan O., Koru M. (2020). Numerical investigation of patient-specific thoracic aortic aneurysms and comparison with normal subject via computational fluid dynamics (CFD). Med. Biol. Eng. Comput..

[46] Leuprecht A., Kozerke S., Boesiger P., Perktold K. (2003). Blood flow in the human ascending aorta: A combined MRI and CFD study. J. Eng. Math..

[47] Hashemi J., Patel B., Chatzizisis Y.S., Kassab G.S. (2021). Study of coronary atherosclerosis using blood residence Time. Front. Physiol..

[48] Pirentis A.P., Kalogerakos P., Mojibian H., Elefteriades J.A., Lazopoulos G., Papaharilaou Y. (2022). Automated ascending aorta delineation from ECG-gated computed tomography images. Med. Biol. Eng. Comput..

[49] Condemi F., Campisi S., Viallon M., Croisille P., Fuzelier J., Avril S. (2018). Ascending thoracic aorta aneurysm repair induces positive hemodynamic outcomes in a patient with unchanged bicuspid aortic valve. J. Bioeng..

[50] Bit A., Alblawi A., Chattopadhyay H., Quais Q.A., Benim A.C., Rahimi-Gorji M., Do H. (2020). Three dimensional numerical analysis of hemodynamic of stenosed artery considering realistic outlet boundary conditions. Comput. Methods Programs Biomed..

[51] Belkacemi D., Abbés M.T., Al-Rawi M., Al-Jumaily A.M., Bachene S., Laribi B. (2023). Intraluminal thrombus characteristics in AAA patients: Non-Invasive diagnosis using CFD. Bioengineering.

[52] Kelsey L.J., Powell J.T., Norman P.E., Miller K., Doyle B.J. (2017). A comparison of hemodynamic metrics and intraluminal thrombus burden in a common iliac artery aneurysm. Int. J. Numer. Methods Biomed. Eng..

[53] Tzirakis K., Kamarianakis Y., Kontopodis N., Ioannou C.V. (2023). The Effect of Blood Rheology and Inlet Boundary Conditions on Realistic Abdominal Aortic Aneurysms under Pulsatile Flow Conditions. Bioengineering.

[54] Belkacemi D., Al-Rawi M., Abbes M.T., Laribi B. (2022). Flow Behaviour and Wall Shear Stress Derivatives in Abdominal Aortic Aneurysm Models: A Detailed CFD Analysis into Asymmetry Effect. CFD Lett..

[55] Peiffer V., Sherwin S.J., Weinberg P.D. (2013). Computation in the rabbit aorta of a new metric–the transverse wall shear stress to quantify the multidirectional character of disturbed blood flow. J. Biomech..

[56] Himburg H.A., Grzybowski D.M., Hazel A.L., LaMack J.A., Li X.M., Friedman M.H. (2004). Spatial comparison between wall shear stress measures and porcine arterial endothelial permeability. Am. J. Physiol. Heart Circ..

[57] Morbiducci U., Gallo D., Ponzini R., Massai D.N.C., Antiga L., Montevecchi F.M., Redaelli A. (2010). Quantitative analysis of bulk flow in Image-Based Hemodynamic Models of the carotid bifurcation: The influence of outflow conditions as test case. Ann. Biomed. Eng..

[58] Achille P.D., Tellides G., Figueroa C.A., Humphrey J.D. (2014). A haemodynamic predictor of intraluminal thrombus formation in abdominal aortic aneurysms. Proc. R. Soc. A Math. Phys. Eng. Sci..

[59] Mutlu O., Salman H.E., Al-Thani H., El-Menyar A., Qidwai U., Yalcin H.C. (2023). How does hemodynamics affect rupture tissue mechanics in abdominal aortic aneurysm: Focus on wall shear stress derived parameters, time-averaged wall shear stress, oscillatory shear index, endothelial cell activation potential, and relative residence time. Comput. Biol. Med..

[60] Ohhara Y., Oshima M., Iwai T., Kitajima H., Yajima Y., Mitsudo K., Krdy A., Tohnai I. (2016). Investigation of blood flow in the external carotid artery and its branches with a new 0D peripheral model. BioMed. Eng. Online.

[61] Curta A., Jaber A., Rieber J., Hetterich H. (2021). Estimation of endothelial shear stress in atherosclerotic lesions detected by intravascular ultrasound using computational fluid dynamics from coronary CT scans with a pulsatile blood flow and an individualized blood viscosity. Clin. Hemorheol. Microcirc..

[62] Cheng H., Zhong W., Wang L., Zhang Q., Ma X., Wang Y., Wang S., He C., Wei Q., Fu C. (2023). Effects of shear stress on vascular endothelial functions in atherosclerosis and potential therapeutic approaches. Biomed. Pharmacother..

[63] Fuchs A., Berg N., Fuchs L., Prahl Wittberg L. (2023). Assessment of Rheological Models Applied to Blood Flow in Human Thoracic Aorta. Bioengineering.

[64] Tzirakis K., Kamarianakis Y., Kontopodis N., Ioannou C.V. (2023). Classification of Blood Rheological Models through an Idealized Symmetrical Bifurcation. Symmetry.

[65] Al-Rawi M., Djelloul B., Al-Jumaily A. (2024). Comparison of Laminar and Turbulent K-Omega Shear Stress Transport Models Under Realistic Boundary Conditions Using Clinical Data for Arterial Stenosis. J. Eng. Sci. Med. Diagn. Ther..

[66] Al-Rawi M. Two-Way Interaction (Aorta Blood-Artery) Using Computational Fluid Dynamics (CFD) Simulation. Proceedings of the IEEE 4th Eurasia Conference on Biomedical Engineering, Healthcare and Sustainability (ECBIOS).

